# Two new cytotypes reinforce that *Micronycteris hirsuta* Peters, 1869 does not represent a monotypic taxon

**DOI:** 10.1186/1471-2156-14-119

**Published:** 2013-12-20

**Authors:** Talita FA Ribas, Luis RR Rodrigues, Cleusa Y Nagamachi, Anderson JB Gomes, Thayse CM Benathar, Patricia CM O’Brien, Fengtang Yang, Malcolm A Ferguson-Smith, Julio C Pieczarka

**Affiliations:** 1 Laboratório de Citogenética, Instituto de Ciências Biológicas, Universidade Federal do Pará, Campus do Guamá, Av. Perimetral, sn. Guamá, Belém, Pará, 66075–900, Brazil; 2 Laboratório de Genética e Biodiversidade, ICED, Universidade Federal do Oeste do Pará, Santarém, Brazil; 3 CNPQ Researcher, Belém, Brazil; 4 Cambridge Resource Centre for Comparative Genomics, University of Cambridge, Cambridge, UK; 5 Cytogenetics Facility, Wellcome Trust Sanger Institute, Cambridge, UK

## Abstract

**Background:**

The genus *Micronycteris* is a diverse group of phyllostomid bats currently comprising 11 species, with diploid number (2n) ranging from 26 to 40 chromosomes. The karyotypic relationships within *Micronycteris* and between *Micronycteris* and other phyllostomids remain poorly understood. The karyotype of *Micronycteris hirsuta* is of particular interest: three different diploid numbers were reported for this species in South and Central Americas with 2n = 26, 28 and 30 chromosomes. Although current evidence suggests some geographic differentiation among populations of *M. hirsuta* based on chromosomal, morphological, and nuclear and mitochondrial DNA markers, the recognition of new species or subspecies has been avoided due to the need for additional data, mainly chromosomal data.

**Results:**

We describe two new cytotypes for *Micronycteris hirsuta* (MHI) (2n = 26 and 25, NF = 32), whose differences in diploid number are interpreted as the products of Robertsonian rearrangements. C-banding revealed a small amount of constitutive heterochromatin at the centromere and the NOR was located in the interstitial portion of the short arm of a second pair, confirmed by FISH. Telomeric probes hybridized to the centromeric regions and weakly to telomeric regions of most chromosomes. The G-banding analysis and chromosome painting with whole chromosome probes from *Carollia brevicauda* (CBR) and *Phyllostomus hastatus* (PHA) enabled the establishment of genome-wide homologies between MHI, CBR and PHA.

**Conclusions:**

The karyotypes of Brazilian specimens of *Micronycteris hirsuta* described here are new to *Micronycteris* and reinforce that *M. hirsuta* does not represent a monotypic taxon. Our results corroborate the hypothesis of karyotypic megaevolution within *Micronycteris*, and strong evidence for this is that the entire chromosome complement of *M. hirsuta* was shown to be derivative with respect to species compared in this study.

## Background

The big-eared bats genus *Micronycteris* Gray 1866 is an antique and diversified lineage of phyllostomids occurring from Mexico to Paraguay and throughout most parts of South America [1]. This lineage diverged from a sister group, *Lampronycteris*, approximately 23.2 million years ago (MYA) [2-5]. The Sanborn review [6] recognized 13 species classified in six subgenera: *Glyphonycteris, Lampronycteris, Micronycteris, Neonycteris, Trinycteris* and *Xenoctenes.* Since then, *Micronycteris lato sensu* has undergone considerable taxonomic changes. The monophyly of *Micronycteris* (*sensu* Sanborn [6]) was not supported by morphological traits, and the fusion of *Xenoctenes* and *Micronycteris* had been proposed [7,8]. Wetterer *et al.*[3] recommended the elevation to genus status for all the *Micronycteris* subgenera, and this has been supported by molecular data [9,10]. They recognized six species: *Micronycteris megalotis*, *M. microtis*, *M. hirsuta*, *M. minuta*, *M. sanborni* and *M. schmidtorum*. Simmons *et al.*[11] recognized two additional species in this genus: *M. brosseti* and *M. homezi*. Lately, three new species have been discovered: *M. matses*[11], *M. giovanniae*[12] and *M. buriri*[4], totaling eleven species in *Micronycteris* (*stricto sensu*).

From external morphology aspects the big-eared bats (*Micronycteris* spp.) can be clustered into two distinct groups: the “dark-bellied” (*hirsuta, matses, megalotis, microtis, giovanniae* and *buriri*) and “pale-bellied” (*brosseti, homezi, minuta, sanborni* and *schmidtorum*) groups [7,11,13]. However, molecular data suggested that “dark-bellied” and “pale-bellied” are not natural groups [13].

In the last three decades, phyllostomid bats have been extensively studied by cytogenetics. Recent advances in molecular cytogenetic methods, that enable genomic mapping and comparison by chromosome painting, have improved greatly our comprehension of karyotypic evolution in mammals. Chromosome painting has been used successfully in the investigation of the evolutionary history of the order Chiroptera. Classical banding techniques together with chromosome painting has allowed the identification of chromosomal rearrangements that have occurred during karyotype evolution of the group and has confirmed the effectiveness of the probes produced for comparative studies of bats [14-25].

Cytogenetic studies in various species of *Micronycteris* show diploid number ranging from 26–40 [12,26-29]. Despite technical advances, the karyotype of *Micronycteris hirsuta* has been poorly characterized with conventional staining [12,26], and with C-banding of sex chromosomes [30,31]. These studies reveal three chromosomal races with distinct karyotypes, namely 2n = 26, FN = 30 (Ecuador), 2n = 28, FN = 32 (Trinidad and Tobago) and 2n = 30, FN = 32 (Honduras, Nicaragua and Suriname). According to Baker *et al.*[26], karyotypes with 2n = 28 and 30 chromosomes differ due to centric fusion events. Molecular data supports high divergence within *M. hirsuta*, corroborating the karyotypic studies [13]. Therefore, it is not clear if *M. hirsuta* represents a monotypic taxon.

Chromosome painting is important on the resolution of phylogenetic and cytotaxonomic questions, and is useful to understand the mechanisms of chromosomal differentiation occurred during the evolution of bats [14-23]. Until now, just six species encompassing few Phyllostomidae subfamilies were studied by ZOO-FISH using probes from *Phyllostomus hastatus* (Phyllostominae) and *Carollia brevicauda* (Carolliinae) [17]: *Desmodus rotundus*, *Diaemus youngi* and *Dyphylla ecaudata* (Desmodontinae) [32], *Artibeus obscurus*, *Uroderma bilobatum* and *U. magnirostrum* (Stenodermatinae) [18]. Two chromosomes were found entirely preserved in karyotypes of these subfamilies: (CBR7 = PHA11 and CBR9 = PHA14), and probably were present of the ancestral karyotype of Phyllostomidae since they are preserved in different species distant phylogenetically. These results leave no doubt that these paints will be useful together for studying the chromosomal relationships among the Phyllostomidae bats.

In order to improve our understanding of karyotypic diversity of *Micronycteris* genus and the chromosomal evolution of Phyllostomid bats, we have analyzed the karyotype of *Micronycteris hirsuta* (Micronycterinae) from the Amazon Forest (Brazil). We used classical banding and comparative genomic mapping with whole chromosome probes from *Carollia brevicauda* (Carolliinae) and *Phyllostomus hastatus* (Phyllostominae). The chromosomal homologies observed were used to infer evolutionary relationships among the different subfamilies of Phyllostomidae.

## Results

### Classic cytogenetic and FISH of telomeric and rDNA 18S probes

**Figure 1:**
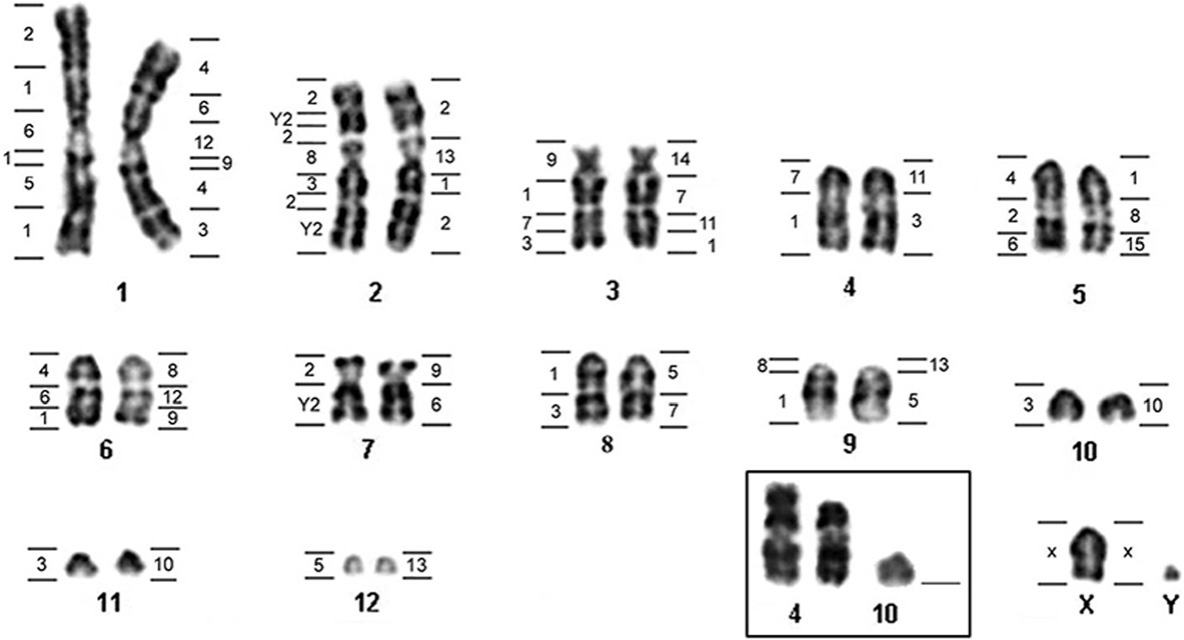
***Micronycteris hirsuta *G-banding karyotype showing regions homologous to *Carollia brevicauda *(left) and *Phyllostomus hastatus *(right).** The boxed chromosomes show the centric fusion between pairs 4 and 10.

**Figure 2:**
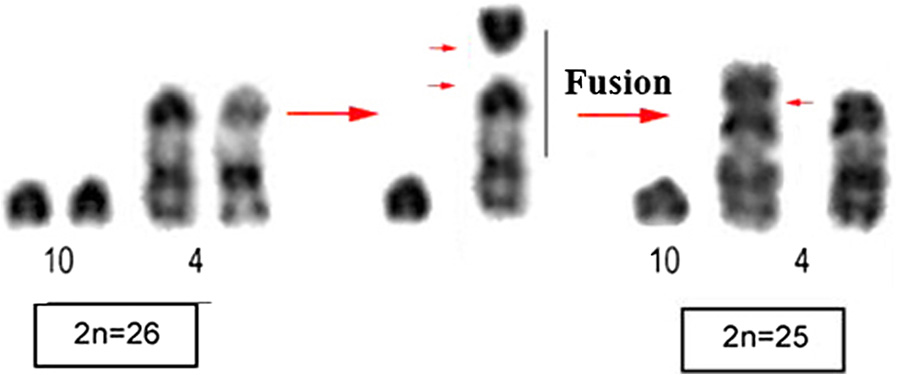
**Robertsonian fusion involved in the origin of chromosomes 4 and 10.** Small red arrows suggest the break points.

**Figure 3:**
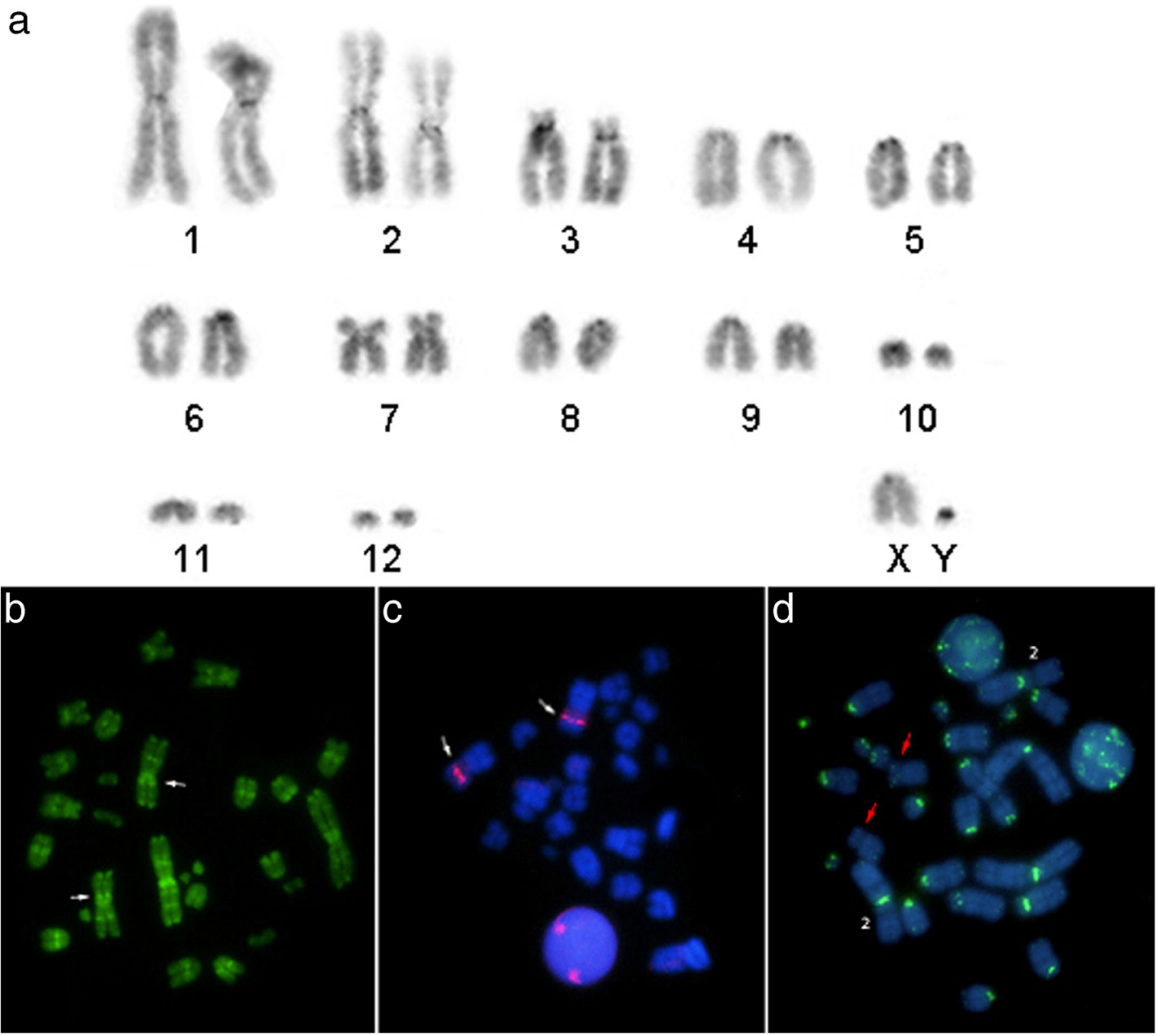
***Micronycteris hirsuta *karyotype and metaphases. a**. C-banding. **b**. CMA_3_ staining. **c**. Partial metaphase of FISH with 18S rDNA probe. **d**. FISH with telomeric probe. White arrows show NOR bearing chromosome 2, and red arrows indicate the chromosome pair which does not show telomeric signals on its centromeric region.

All *Microncyteris hirsuta* samples have a diploid number of 2n = 26, FN = 32, except for the specimen LR-275 that had a 2n = 25 karyotype. In the karyotype with 2n = 26, two chromosomal pairs are metacentric, one pair is submetacentric, one pair is subtelocentric and eight pairs are acrocentric (Figure 1; Table 1). The sex system is simple (XX/XY) and the sex chromosomes are acrocentric, with a small Y. The karyotype with 2n = 25 was heterozygous for a centric fusion rearrangement involving chromosomes 4 and 10 (Figure 2). C-banding detected the presence of small amounts of constitutive heterochromatin in the centromeric region of all chromosomes (Figure 3a).

**Table 1:** **Karyotypic data of *Micronycteris hirsuta***

**Locality**	**2n**	**NF**	**Chromosomal morphology**			**Reference**
			**Acro**	**Subtelo**	**Meta/Sub**	
Itaituba-Brazil	25	32	14	2	7	This paper
Itaituba-Faro-Brazil	26	32	16	2	6	This paper
Ecuador	26	30	18	2	4	Fonseca *et al.,* 2007 [12]
Trinidad and Tobago	28	32	20	2	4	Baker *et al.,* 1973 [26]
Honduras, Nicaragua and Suriname	30	32	24	2	2	Baker *et al.,* 1973, 1981; Baker, 1979 [26,27,29]

Staining with AgNO_3,_ CMA3 and FISH with 18S rDNA probes revealed a Nucleolar Organizer Region (NOR) in the middle of the short arm of chromosome 2 (Figure 3c). Telomeric probes hybridized to the centromeric regions of all chromosomes, except the smallest submetacentric. Weak hybridization signals could be visualized on the telomeric region of a few chromosomes (Figure 3d).

### Hybridization of *Phyllostomus hastatus* probes onto *Micronycteris hirsuta*

Hybridization of *Phyllostomus hastatus* (PHA) whole chromosome probes revealed 32 homologous segments on the *M. hirsuta* (MHI) genome (Figure 1). Three *Phyllostomus* probes (PHA-14, 15 and X) give just one fluorescent signal on the chromosomes of *Micronycteris,* corresponding to segments of MHI-3, MHI-5 and entirely on to X, respectively.

Eight paints of *Phyllostomus* yielded two hybridization signals, with each probe marking regions of two distinct chromosomes in *Micronycteris*: PHA-3 (MHI-1 and 4), PHA-5 (MHI-8 and 9), PHA-6 (MHI-1 and 7), PHA-7 (MHI-3 and 8), PHA-8 (MHI-5 and 6), PHA-10 (MHI-10 and 11), PHA-11 (MHI-3 and 4) and PHA-12 (MHI-1 and 6).

**Figure 4:**
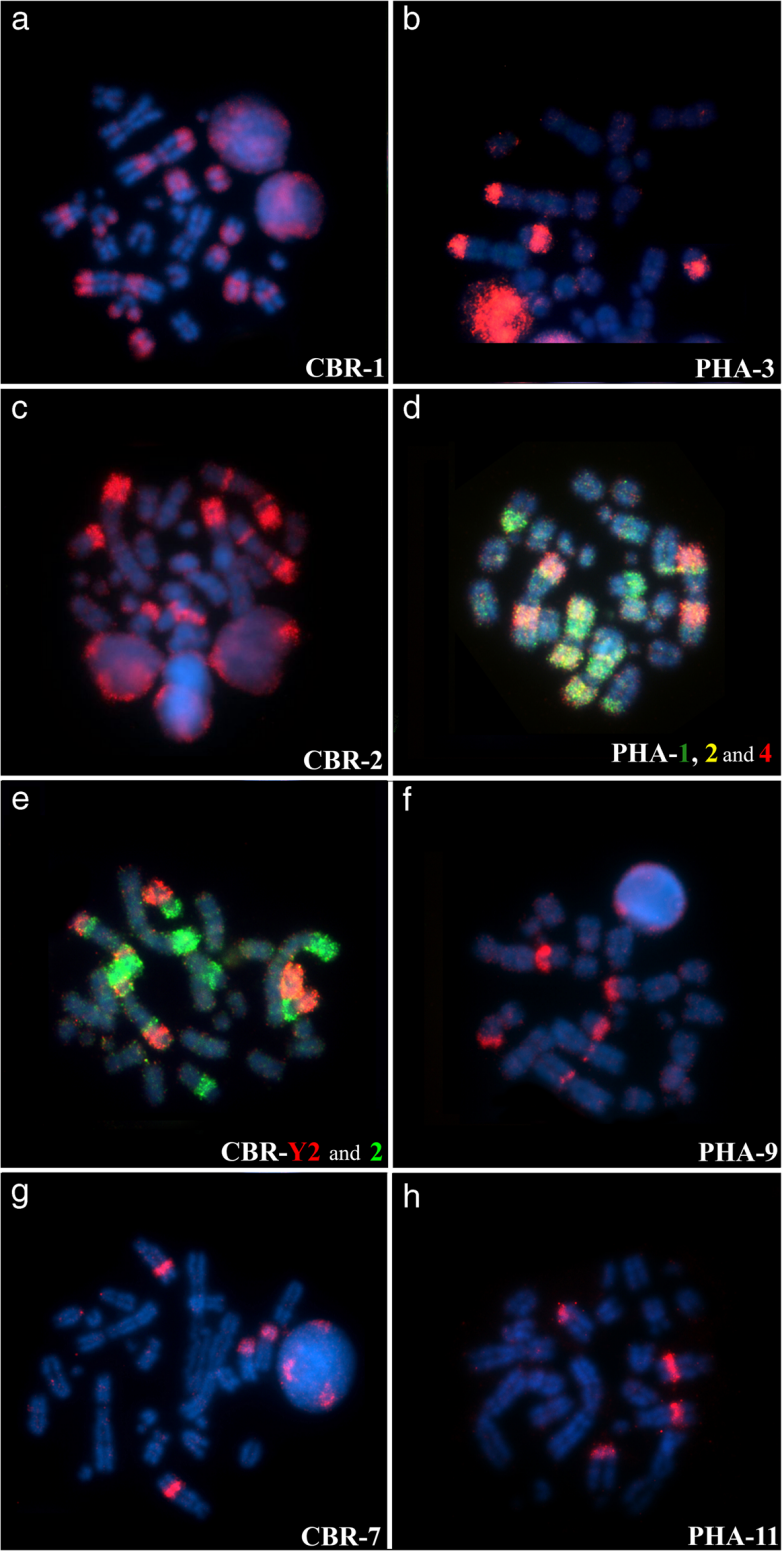
**Examples of chromosomal painting in *Micronycteris hirsuta, *** **using probes of CBR (left), and probes of PHA (right) onto: a) CBR-1 (pairs 1, 3, 4, 6, 8 and 9); b) PHA-3 (pairs 1 and 4); c) CBR-2 (pairs 1, 2, 5 and 7); d) PHA-1, 2 and 4 (pairs 1, 2, 3 and 5); e) CBR-Y2 and 2 (pairs 1, 2, 5 and 7); f) PHA-9 (pairs 1, 6 and 7); g) CBR-7 (pairs 3 and 4); h) PHA-11 (pairs 3 and 4).**

Two PHA paints showed two signals, but hybridized to parts of just one chromosome in *Micronycteris*: PHA-2 (MHI-2) and PHA-4 (MHI-1). Three paints of *Phyllostomus* each hybridized to three chromosomes of *Micronycteris*: PHA-1 (MHI-2, 3 and 5), PHA-9 (MHI-1, 6 and 7) and PHA-13 (MHI-2, 9 and 12) (Figure 1). The Figure 4(b, d, f, h) shows some hybridization with PHA probes on metaphases of MHI.

### Hybridization of *Carollia brevicauda* painting probes onto *Micronycteris hirsuta*

Comparative painting with *Carollia brevicauda* (CBR) probes revealed 35 homologous segments on the *Micronycteris hirsuta* (MHI) genome (Figure 1). Two paints of *Carollia* gave just one signal of hybridization on MHI metaphases: CBR-9 hybridized on the short arm of MHI-3 and CBR-X entirely hybridized on MHI-X.

Four paints of *Carollia* each hybridized to two *Micronycteris* chromosomes: CBR-4 (MHI-5 and 6), CBR-5 (MHI-1 and 12), CBR-7 (MHI-3 and 4) and CBR-8 (MHI-2 and 9). CBR-Y2 hybridized to two chromosomes, but showed three hybridization signals, two on MHI-2 and one on MHI-7.

CBR-2 hybridized to chromosomes MHI-1, 2, 5 and 7, showing three fluorescents signals on chromosome 2, and gave just one signal on other chromosomes. CBR-3 hybridized to five distinct segments on MHI chromosomes: 2, 3, 8, 10 and 11. CBR-1 hybridized to 8 regions over six different chromosomes: MHI-1 (three signals), 3, 4, 6, 8 and 9. The Figure 4(a, c, e, g) shows some hybridization with CBR probes on metaphases of MHI. The results of complete mapping of PHA and CBR whole chromosome probes on G-banded karyotype of MHI are on Figure 1.

## Discussion

### Karyotypic diversity in *Micronycteris hirsuta*

Here we describe for the first time cytogenetic data of *Micronycteris hirsuta* from the Amazon Region, Brazil. Specimens presents distinct karyotypes with 2n = 25 (7 M/SM + 2ST + 14A) and 26 (6 M/SM + 2ST + 16A) chromosomes, FN = 32 in both. The difference in diploid number is explained satisfactorily by a fusion involving chromosomes 4 and 10 (Figure 2). These karyotypes differ from those previously reported for this species [12,26,27,29], where the same type of rearrangement found here is believed to explain in part the karyotypic diversity observed in *M. hirsuta*.

The distribution pattern of constitutive heterochromatin in only a few chromosomes differs from that in *Lampronycteris brachyotis* (Micronycterinae) [28], where the entire chromosome complement presents conspicuous markings. This result found in *Micronycteris hirsuta* is in agreement with previous observations that bats have a strong tendency to reduction in genomic size (or C-value) and consequently a decrease of regions containing highly repetitive DNA [33,34].

The NOR was found interstitially in one chromosome pair in all specimens of *Micronycteris hirsuta* studied here. This same chromosome can be easily identified by the presence of a secondary constriction in specimens of *M. hirsuta* studied in Ecuador with 2n = 26 chromosomes [12]. The NORs labeled positive for fluorochrome CMA_3_, but negative for DAPI, indicating that the ribosomal DNA is interspersed with repetitive GC-rich DNA.

In addition to signals in telomeric regions, the telomeric probe produced unexpected signals in the centromeric regions of chromosomes in *Micronycteris hirsuta*. The intrachromosomal or interstitial telomeric sequences (ITSs) may correspond to sequences of repetitive DNA similar to telomeric sequences, but probably are not originating from the telomeres, as evidenced in other species of bats [35-37].

Considering that all species of the genus *Micronycteris,* except *Micronycteris hirsuta,* have a bi-armed X chromosome, it is likely that the acrocentric shape of the X is an autapomorphic character of this species, as previously suggested by Rodrigues *et al.*[38] and Noronha *et al.*[39]. In contrast, the primitive condition of X for phyllostomids would be a bi-armed form as found in *Macrotus waterhousii*, *Phyllostomus discolor* and *P. hastatus*[28,29,38,40].

Table 1 summarizes the karyotypic data of *Micronycteris hirsuta* analyzed to date. Comparing karyotypes studied here and those from Central and South America with 2n = 28 (Trinidad and Tobago) [26] and 30 chromosomes (Honduras, Nicaragua and Suriname) [26,27], the differences can be explained by two Robertsonian rearrangements (fusion/fission) between acrocentric chromosomes. Despite their similar diploid number (2n = 26) [12], populations from Ecuador and Brazil diverge by three rearrangements (2 fusion/fission and 1 pericentric inversion) resulting in morphological alterations of five chromosome pairs (1, 2, 5, 7 and 8 in Figure 1, present study). When compared with the 2n = 28 karyotype from Trinidad and Tobago, two rearrangements for the Ecuadorian population and a single for the Brazilian population are enough to explain their divergence. Therefore, we assume that karyotypes with 2n = 26 from Ecuador and Brazil may have evolved independently starting from an ancestral with 2n = 28.

The sharing of the NOR-bearing pair in the karyotype of specimens from Brazil and Ecuador (see Figure seven in reference [12]), reinforces the argument that karyotypic divergence occurred by centric fusion rearrangements that led to a reduction in the diploid numbers. The similarity between the specimens from Brazil and Ecuador is best explained by karyotypic homoplasy on the diploid number and it is highly likely that the karyotype 2n = 28 chromosomes represents the ancestral karyotype these cytotypes. Thus, karyotypic data suggest that the specimens of Trinidad and Tobago (2n = 28) are closer to the Brazilian (2n = 26, NF = 32) and equatorial (2n = 26, NF = 30) specimens.

**Figure 5:**
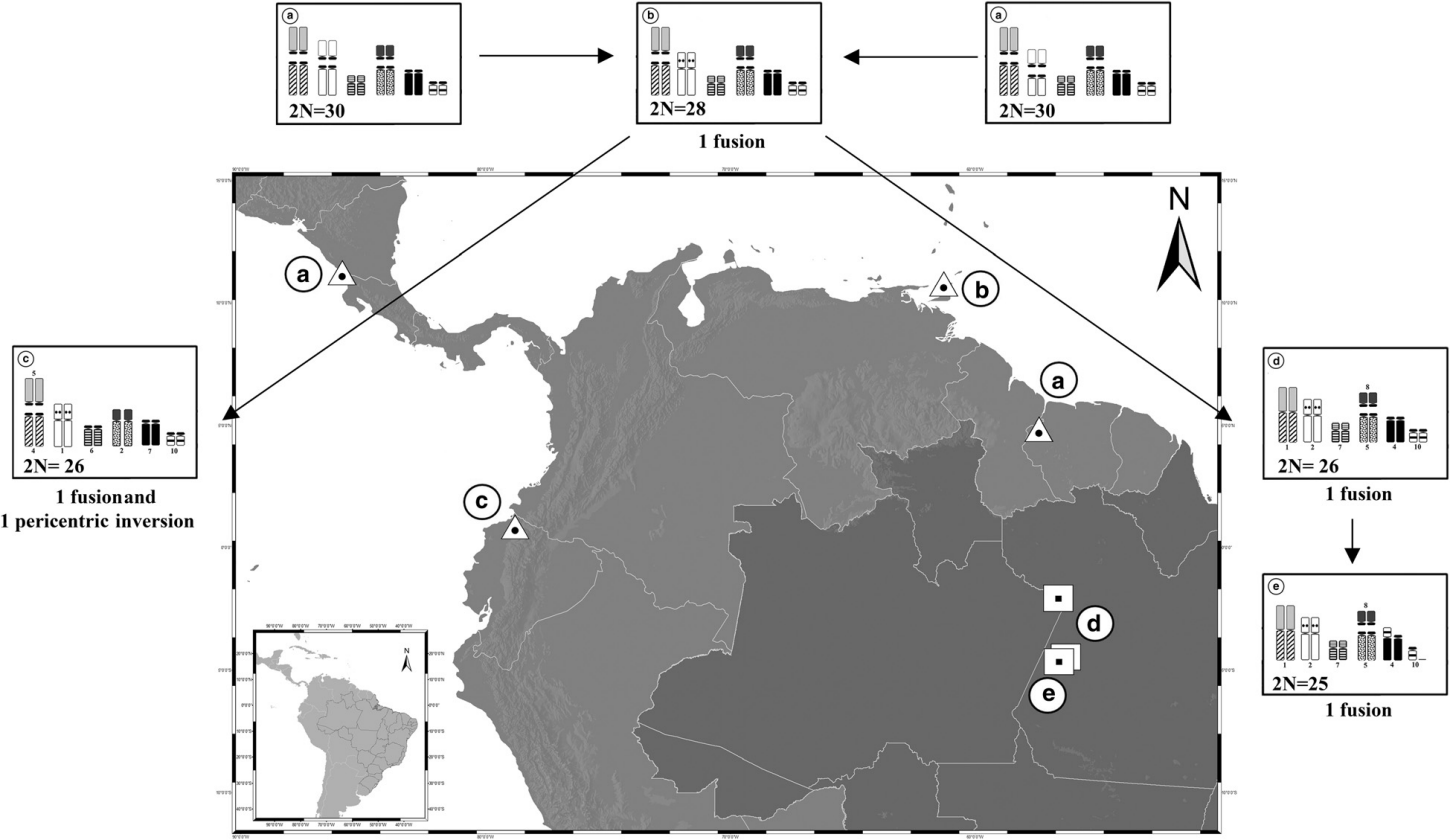
**Map of compared samples of *Micronycteris hirsuta.*** Triangles indicate the sites from which previous cytogenetic descriptions were performed. Squares represent the cytogenetic samples studied herein (see Table 1 for locality details). Idiograms plotted beside each collection site represent the unique or shared chromosomes among specimens. Diploid number (2n) of specimen and the rearrangements that differentiate the different cytotypes are indicated below each idiogram. Idiograms of MHI from **a)** MHI from Honduras, Nicaragua and Suriname; **b)** Trinidad and Tobago; **c)** Ecuador; **d)** and **e)** Present work. For specimens of Ecuador with 2n = 26 the numeration of chromosome arms is according Fonseca *et al.*[12]. For details see text and Table 1.

Moreover the Andean Cordillera, a geographic barrier that crosses the center of Ecuador from the north to south, has probably contributed to isolating populations from Brazil and Ecuador. This hypothesis is plausible considering that the events which led to the diversification of the genus *Micronycteris* occurred during the Pliocene and Pleistocene epochs (5.0 and 0.6 million years ago) and the corresponding lineage of *M. hirsuta* that diversified about 1.0 million years ago (range 2.0 to 0.5 MYA) [4], while the uplift of the Andes was completed about 2.5 Million years ago during the Quaternary [41]. A tentative interpretation of karyotypic differentiation routes in *M. hirsuta* is shown in Figure 5, where examination of karyotypes seems to indicate that geographic variation of diploid numbers exhibits a decrease from North to South.

From Figure 5 we note that there are different diploid numbers in different locations but there is no information on the intermediary regions. Two possibilities can be suggested: 1) If intermediary karyotypes are found in the intermediary regions, this would be a typical case of chromosomal polymorphism. 2) If there are no intermediary karyotypes, the different populations with different diploid numbers are reproductively isolated, meaning that they are different species and *Micronycteris hirsuta* would not represent a monotypic taxon. Under this view, the karyotype with 2n = 25 (LR-275) can have three explanations: 1) this would be a single heterozygous specimen found as result of a balanced rearrangement Robertsonian (fusion) that occurred during gametogenesis of one of its parents. 2) An intermediary karyotype that is probably common, meaning that this is a chromosomal polymorphism. 3) An evidence of a hybrid zone where the heterozygote sample with 2n = 25 was a hybrid derived from a cross between two homozygous forms with 2n = 26 and 24 chromosomes (the latter remains to be found).

Analyses of mitochondrial (cytochrome b) and nuclear (intron 7 of the nuclear fibrinogen) genes demonstrate that populations of *M. hirsuta* are subdivided into three distinct clades [4,13]. Ecuadorian specimens with 2n = 26 are phylogenetic closer with populations of Panama (diploid number unknown). On the other hand, specimens of Trinidad and Tobago with 2n = 28 are more similar of phylogenetic standpoint with specimens collected in French Guiana (diploid number unknown) [4,12,13]. Although these data indicate a phylogenetic proximity between populations of Panama and Ecuador, the karyotypic data available are yet incipient to corroborate these phylogenetic relationships. In order to clarify this issue, obtaining cytogenetic data of intermediate populations across the geographic distribution of *M. hirsuta* that may reveal new karyotypic forms, and analysis of nuclear and mitochondrial genes are sorely needed.

The cytotypes described here, added to evidence suggesting some geographic differentiation of populations based in karyotypic, mitochondrial, nuclear, and morphological markers [26], strongly suggest that *Micronycteris hirsuta* do not represent a monotypic taxa.

Additionally, the record of *Micronycteris hirsuta* in two localities of eastern Amazonia described here is an increase in the distribution of this species in South America to about 700 km. Although rarely captured, species of big eared-bats represent a common component of Neotropical rain forests [11]. Therefore it is possible that the occurrence of *M. hirsuta* in Brazil and the Amazon region have been underestimated, since there are records of this species in the Atlantic Forest [42].

### *Micronycteris hirsuta* and its correlation with Phyllostomidae

Recent data from chromosome painting, associated with G-banding, confirm that Robertsonian fusions with the complete conservation of chromosome or whole arms, and inversions are the main mechanisms of karyotypic differentiation in the Order Chiroptera [19,20,22,24,25,32]. Herein we found conservatism in many chromosomal segments shared between *Micronycteris hirsuta*, *Phyllostomus hastatus* and *Carollia brevicauda*, but homology of whole chromosomes was not detected in the autosomal set.

A comparison of *Micronycteris hirsuta* painted chromosomal map with other phyllostomids previously investigated [17,18,32], revealed that many rearrangements have occurred in the differentiation of the *M. hirsuta* karyotype from the phyllostomid common ancestor. Even the highly conserved syntenic group (PHA-11; CBR-7), shared by *Phyllostomus*, *Carollia, Artibeus, Uroderma magnirostrum*[17,18] and Desmodontinae bats [32], was broken in the MHI genome (MHI-3 and MHI-4) (Figure 1). In the other hand, although not broken, the chromosome PHA-14 is fusioned with PHA-11 in MHI, with PHA-9 in UBI and, in the Desmodontinae bats with two distinct chromosomal segments: 12 in DYO and 13 in DRO [32].

These chromosomes have been suggested to be present on the ancestral karyotype of the family Phyllostomidae, since it was present and conserved in the karyotype of such phylogenetically distant species of uncorrelated subfamilies mapping so far [17,18,32]. Although MHI represent a basal clade for Phyllostomidae, and has previously been suggested that basal taxa could contribute to confirm the chromosomal ancestrality [32], we argue that not necessarily basal taxa have primitive karyotypes. It is more probably that these chromosomes forms found in MHI correspond to derivative forms within Phyllostomidae and autoapomorphies in this species, without phylogenetic value.

Classical cytogenetic studies had already suggested a high rate of karyotype evolution within the big-eared bats lineage [28,43]. Arnold *et al.*[44] argue that, compared to their congeners, *Micronycteris hirsuta* has undergone extensive and independent karyotypic changes, which has been supported by other authors [3,12,13]. Our results corroborate the hypothesis of karyotypic megaevolution within *Micronycteris*, and strong evidence for this is that the entire chromosome complement of *M. hirsuta* proved to be derivative with respect to species compared in this study.

## Conclusions

In conclusion, the karyotypes of Brazilian specimens of *Micronycteris hirsuta* (2n = 26 and 25, FN = 32), show variation in both diploid and fundamental number, in relation to specimens from Central and South America, and therefore are new karyotypic forms reported to this species. The entire autosomal chromosome complement of *M. hirsuta* seems to be highly derived in relation to that of other phyllostomid bats, and the finding of new cytotypes reinforce that *Micronycteris hirsuta* does not represent a monotypic taxon.

## Methods

### Specimens analyzed

Six specimens (four females and two males) of *Micronycteris hirsuta* were collected from natural populations during field expeditions to assess biodiversity in the Amazon Region, Brazil. The study sites were: Itaituba (S04°28′20, 5”/W56°17′03,7) and Faro (S02°04′43,4”/W 56°37′12,0”), municipalities of Pará, Brazil (Table 1). *Voucher* specimens (LR-194, LR-275, LR-1342, LR-2104, LR-2160 and LR-2428) were fixed in 10% formalin preserved in 70% ethanol and deposited in the mammal collection of the Museu Paraense Emilio Goeldi and Museu de Zoologia da Universidade Federal do Oeste do Pará.

### Chromosomal preparation, chromosomal banding and staining with fluorochromes

Chromosomal preparations were obtained by direct extraction from bone marrow after Colchicine treatment following Baker *et al.*[45]. Conventional staining was used for diploid (2n) and fundamental numbers (FN) determination. G-banding followed two distinct methods: trypsin treatment [46], and saline solution (2xSSC) incubation [47]. In both methods the metaphases were stained with Wright’s solution. C-banding was carried out according to Sumner [48] and Ag-NOR staining followed Howell and Black [49]. For observation of GC/AT base pairs rich regions, metaphase chromosomes were stained with CMA_3_ following Schweizer [50] and DAPI according to Pieczarka *et al.*[51], both with modifications.

### Fluorescence *in situ* hybridization (FISH)

FISH with digoxigenin labeled telomeric probes (All Human Telomere Probes, Oncor) were performed according to the manufacturer’s protolocol and 18S rDNA probes from *Prochilodus argenteus*[52], were labeled with biotin or digoxigenin by nick translation. Chromosome-specific painting probes from *P. hastatus* and *C. brevicauda* were generated from flow-sorted chromosomes and labeled by DOP-PCR (degenerate oligonucleotide-primed-polymerase chain reaction) amplification [53], and used to determine chromosomal homologies in *M. hirsuta*. The chromosome painting experiments were carried out following procedures previously described [17,54]. Briefly, the slides were incubated in pepsin solution, and dehydrated in an ethanol series (70, 90 and 100%), air-dried and aged in a 65°C incubator for two hours. Chromosomal DNA was denatured in 70% formamide/2xSSC for 40 seconds, and the slides immersed immediately in cold 70% ethanol for 4 minutes. For single-color detection, biotin-labeled probes were visualized with Cy3-avidin or FITC-avidin. For two-color detection, Cy3-labelled probes were used together with biotin-labeled probes in the same experiment. After hybridization and washing, the metaphases were stained with DAPI. Images were captured using the Axiovision 3.0 software with a CCD camera (Photometrics C250/A) coupled on a Zeiss-Axiophot 2 microscope or with a software Nis-Elements on a Nikon H550S microscope. For image processing Adobe Photoshop CS4 software was used.

The karyotypes of Desmodontinae bats [32], were used in the comparative analysis. The chromosome complement of *Phyllostomus hastatus* (2n = 32) was used as a reference for defining syntenic associations among the compared species, since previous G-banding results indicated that its karyotype retained most of the segments and the supposed ancestral chromosome arms of Phyllostomidae [28].

## Abbreviations

CBR: *Carollia brevicauda*; CMA3: Chromomycin A_3_; DAPI: fluorochorme 4′-6′-diamidino-2-phenylindole; DRO: *Desmodus rotundus*; DOP-PCR: Degenerate oligonucleotide-primed-polymerase chain reaction; DYO: *Diaemus youngi*; FISH: *Fluorescence In Situ Hybridization*; FN: Fundamental number; PHA: *Phyllostomus hastatus*; MHI: *Micronycteris hirsuta*; SSC: Saline sodium citrate.

## Competing interests

The authors declare that they have no competing interests.

## Authors’ contributions

TFAR carried out classic cytogenetic and chromosome painting, organized undertook the bibliographic review, analyzed the data and wrote the paper. LRRR collected most the samples, helped in classic and molecular cytogenetic analysis and contributed to the discussion of data. CYN participated of the techniques development and contributed to the discussion of data. TCMB participated of the techniques development and carried out the FISH with telomeric and rDNA 18S probes. AJBG collected some samples, participated of the techniques development, helped in classic and molecular cytogenetic analysis and contributed to the discussion. PCMO carried out the whole chromosome probes from *Carollia brevicauda* and *Phyllostomus hastatus*. MFS revised the manuscript and contributed to the discussion of data. FY revised the manuscript and participated of the techniques development. JCP coordinated the study and contributed to the discussion. All authors read and approved the final version of the manuscript.
